# How Social Identity Affects Green Food Purchase Intention: The Serial Mediation Effect of Green Perceived Value and Psychological Distance

**DOI:** 10.3390/bs13080664

**Published:** 2023-08-09

**Authors:** Can Zheng, Shuai Ling, Dongmin Cho

**Affiliations:** 1 Department of Design and Manufacturing Engineering, Jeonbuk National University, Jeonju 54896, Republic of Korea; canzheng@jbnu.ac.kr; 2 Department of Industrial Design, Jeonbuk National University, Jeonju 54896, Republic of Korea; mellgipson@jbnu.ac.kr

**Keywords:** green food, social identity, green perceived value, psychological distance, purchase intention

## Abstract

As the global population continues to grow, the impact of environmental damage and resource depletion has been severely increased. In this context, green food gains tremendous potential as a sustainable solution. This study establishes a model framework around social identity, psychological distance, green perceived value, and purchase intention from the perspective of social identity to explore the impact the social group has on individual green food purchase intention. Data from 497 questionnaires collected in China were validated using SPSS26 and SmartPLS4. The results demonstrated that the model exhibited excellent explanatory power for psychological distance (R^2^ = 47.5%), green perceived value (R^2^ = 48.2%), and purchase intention of green food (R^2^ = 54.7%). Path analysis showed that social identity, psychological distance, and green perceived value significantly positively affected green food purchase intention. The results also show that social identity significantly positively affected psychological distance and green perceived value, while psychological distance has a significant positive influence on green perceived value. Additionally, it is concluded that psychological distance and green perceived value have significant mediating and serial mediating effects on social identity and green food purchase intention. These findings bridge the research gap concerning consumers’ green food purchase intention from a group perspective, thereby offering great insights for the formulation of sustainable policies. Furthermore, the study provides both theoretical and practical implications for the expansion of the green food consumption market.

## 1. Introduction

As the economy continues to develop and income levels rise, high-quality food has become a major concern for certain consumers [1,2,3]. Especially due to the appearance of food safety issues across the globe, consumer demand for good quality green food has become more urgent [4]. Taking this into account, healthy and environmentally friendly green food consumption is expected to become the new consumption standard [5]. Green food is a type of food whose production is based on sustainable development principles and is certified by a specialized agency, meeting the production requirements of green, pollution-free, and nutritious food [6]. To receive certification and the green food mark, green food production must meet specific criteria during the production area assessment, production process, product quality, and packaging standards. The mark is protected by trademark law and regulated by the relevant authorities [7]. As reported by the 2018 China Green Food Statistics Annual Report, the national green food industry included 13,203 green food companies and supplied 30,932 green products on 10 December 2018, with a 21.18% and 20.74% year-on-year increase, respectively. The total output of green food for the year was 100,064,400 tons, which shows that the industry has good momentum [8]. China’s green food industry sales experienced fluctuations from 2011 to 2021, followed by a consistent upward trend after 2016. By 2020, green food sales surpassed 500 billion yuan, and exports exceeded 3.6 billion U.S. dollars. In 2021, the sales reached 521.86 billion yuan, marking an increase of 14.29 billion yuan compared to 2020, with a year-on-year growth rate of 2.82% [9]. Green food sourcing that is consistent with sustainable development guidelines cannot only alleviate the resourcing challenges communities face but also tackle environmental problems [10]. Additionally, it can create new opportunities for rural economic development and increase the income of farmers in those areas [11]. Therefore, it is important to study the factors that influence consumer intentions to purchase green food, and thus, promote green food consumption.

Scholars have conducted extensive research on the purchase intentions of green food. Woo et al., used perceived value to explain the formation of green food purchase intention [12]. Xin Qi et al. demonstrated the applicability of models such as the ‘A-TPB’ (Amended Theory of Planned Behavior) model to Chinese consumers’ green food purchase intentions [13]. Wang et al., validate the ‘REB’ (Responsible Environmental Behavior) model based on green food purchases by examining the elements that influence customers’ purchasing intentions [14]. Yogananda et al., demonstrate the substantial impact of environmental concerns, perceived behavioral control, health consciousness, and subjective norms on the purchasing behavior of green food among Malaysian consumers [15].

While scholars have extensively researched green food in the past, the majority of these studies have relied on existing theoretical models, neglecting to explore the influence of social groups on green food purchase intentions. The concept of reference groups posits that individuals’ values, attitudes, and behaviors are both directly and indirectly influenced by the groups they belong to [16]. Consequently, this study places its focus on examining the impact of social groups on green food purchase intentions. The theory of ‘social identity’ is an important psychological theory that explores the influence social groups have on individual consumers [17,18]. Multiple studies have suggested that there is a close association between social identity and consumer purchase intentions [19,20,21]. Therefore, it is crucial to take social identity into account in the discussion of green food consumption and consumer purchase intentions. Social identity refers to “a certain emotional and value-based significance, attributed to an individual by the specific social group to which they belong” [22]. This emotional and value-based meaning can potentially influence consumers’ purchasing behavior in specific circumstances or occasions. Meanwhile, previous studies have overlooked the significant impact of green perceived value and psychological distance on consumer behavior, as well as their role in individual social identification and green food purchase intention. Green perceived value is a theory developed from the theory of perceived value and constitutes an important antecedent that encourages consumers to buy more green food [23,24]. Psychological distance refers to one’s subjective perception of something’s proximity to oneself [25]. It also involves an individual’s subjective judgment of their acceptance [26]. In the marketing sector, it is widely acknowledged that consumers’ perception of psychological distance can significantly impact their cognitive and decision-making processes [27,28,29]. Therefore, psychological distance, like social identity, significantly impacts consumers from the perspective of influencing cognition, whereas green perceived value affects consumers from a comprehensive evaluation standpoint. However, no previous research has integrated the effects of social identity, psychological distance, and green perceived value on consumers’ purchase intention of green food. As a result, this study seeks to establish a model of green food purchase intention under the group perspective by integrating the aforementioned theories.

This study aims to establish a model of consumers’ green food purchase intention from a group perspective. More specifically, this paper includes three main objectives:

1. Study whether the psychological distance and green perceived value can influence consumers’ purchase intention of green food under the group perspective
2. Explore the causal sequence relationship of this influence process
3. Evaluate whether establishing this model can provide better theoretical and practical support for green food purchase intention

Based on these objectives, this study aims to address the following research questions:

1. What is the effect of social identity on consumers’ psychological distance and green perceived value?
2. What is the effect of consumers’ psychological distance and green perceived value on consumers’ green food purchases?
3. Is there a mediating relationship between consumers’ psychological distance and green perceived value between social identity and green food purchase?

To solve these problems, this study established the research model of “social identity—psychological distance—green perceived value—purchase intention”. The data collected from the questionnaires were analyzed with descriptive statistics by SPSS26, and the explanatory and predictive power of the model was tested by using SmartPLS4. The test verified further the relationship between the variables in the model and the mediating role of the two intermediate variables in the model path. Finally, the results of the data analysis are discussed. The results of this study successfully bridge the gap in research on green food purchase intention from a group perspective and contribute to the practical application of related theories in the marketing domain. Additionally, these findings offer a unique perspective for shaping marketing strategies in green food-related industries and effectively boosting consumers’ purchase intention of green food through the influence of social groups. This, in turn, contributes to the sustainable development of green food initiatives.

The structure of this paper comprises seven different parts. The paper begins by providing a brief introduction to the concepts and data related to the green food industry, along with the emphasized theories addressed in the study. Then, the research model and hypotheses of the paper are presented in connection with a comprehensive literature review of the emphasized theories. Following that, statistical methods are employed for data analysis. The hypotheses of the paper are then tested and validated through data analysis. Subsequently, the results are discussed in relation to the literature. The theoretical and practical implications of this study are described. The paper further addresses its research limitations and proposes future research directions for this field of study.

## 2. Research Hypothesis and Theoretical Framework

### 2.1. Research Hypothesis

#### 2.1.1. Social Identity and Purchase Intention

Social identity theory refers to individuals’ definitions of themselves based on two social levels: social category and group membership [30]. This theory is important for exploring the link between individual identity and social groups [31]. Social identity theory is a description of individual characteristics [32] which derives from an individual’s intention of identifying themselves with one or more social groups [33]. Furthermore, existing research indicates that social identity motivates individuals to participate in activities relevant to their chosen social identities [34]. Academics have conducted extensive research on social identity theory. For instance, in this study, Ellemers segmented the dimensions of social identity into single-dimensional components [35]. Additionally, Johnson in his study of social identity, divided it into double-dimensional components, including the cognitive and emotional ones [36]. On the other side, Jenkins divides social identity into intrinsic identity, which refers to the subjective identification of group members with a group, and extrinsic identity, which refers to the social categorization of a group or group [37]. In a later study, Ellemers redefined social identity and based it on three components: cognitive, emotional, and evaluative identity [38]. Ellemers’ research has significantly contributed to future studies [39,40,41]. Cognitive identity refers to the sense of group identification for consumers when they find similarities between their group’s characteristics and those of a different group [42]. For individuals, cognitive identity is seen as a form of self-categorization. Emotional identity is considered to be an individual’s commitment and emotional investment in the group, and evaluative identity is considered relevant to the positive or negative value of group membership [38]. In summary, this paper argues that consumer green food purchase intention should be studied from the perspective of cognitive, emotional, and evaluative social identities.

The existing literature suggests that consumers’ identity impacts their product evaluation, thereby increasing their purchase intention for products associated with social identity information [43,44]. This value linked with group consciousness represents the evaluative aspect of identity within the social identity framework. Emotional identity plays the most significant role in determining consumers’ behavioral preferences when it comes to group identity [38]. Consequently, when consumers have an emotional attachment to a particular group, they tend to align their behaviors with that group’s habits and values. Similarly, cognitive identity motivates consumers to seek self-definition [45] and strategic use of some products to make identity cues [46]. At the same time, cognitive identity refers to the self-categorization of individual members in the consumer’s social group. Therefore, emotional and evaluative identities have been built upon cognitive identity and influence consumer behavior [32]. Studies on the topic of food have shown that the social identity of organic consumer groups has a significant impact on individuals’ intentions to purchase organic food [47]. Therefore, this study expects that consumers’ social identifications closer to green consumer groups will positively influence their purchase intentions toward green food. The hypotheses of this study are as follows:

**Hypothesis** **1a (H1a).** *Cognitive identity positively influences the purchase intention of green food consumers.*

**Hypothesis** **1b (H1b).** *Emotional identity positively influences the purchase intention of green food consumers.*

**Hypothesis** **1c (H1c).** *Evaluative identity positively influences the purchase intention of green food consumers.*

#### 2.1.2. Social Identity and Green Perceived Value

Perceived value is the consumer’s combined evaluation of product value and cost [48]. The term ‘perception’, as emphasized in the above definition, signifies the influence of subjective and objective factors upon the evaluation of value. This can also be explained in Holbrook’s view which suggests that individual variability affects the composition of individually perceived value [49]. Since green food consumption started becoming more popular, many scholars have explored the concept of ‘green perceived value’. Chen et al., and Ariffin et al., describe it as a ‘comprehensive evaluation of green products by consumers’ after comparing the perceived environmental benefits and costs included in the green consumption process [23,50]. As research in this area continues to progress, scholars have primarily focused on delineating green perceived value theories from both one-dimensional and multidimensional perspectives [12,51,52]. Overall, this study describes green perceived value as the comprehensive evaluation of green food after comparing its benefits for the consumers, such as environmental protection and nutritional value, during the purchase process, with the costs, such as time, money, and effort green food consumption includes.

Existing research suggests that individuals are given feedback regarding their social group presence when they better identify with the group [53]. When this feedback is positive, consumer impression is described as happiness or joy [54,55,56]. Based on the previous paragraph, as the level of social identity increases, group cohesion also becomes stronger, and the conversion rate of this feedback is higher. In the field of research on social identity and consumer perceived value, brand identity is demonstrated as an antecedent of consumer perceived value [57]. Studies have shown that if a social group deems a brand trustworthy, then this brand becomes part of the group’s value and is passed on to the individuals to whom the group belongs [58]. Additionally, based on the principle of identity association, when a product becomes associated with a particular social group, consumers’ emotions and attitudes towards that group are likely to influence their choices and evaluations of the product. This can lead to more favorable and positive evaluations when the product is congruent with the consumer’s group identity [59]. According to the ‘meaning transfer theory’, individuals can achieve “self-expression” by transferring meaning from more influential individuals, groups, or symbols to themselves to obtain the desired value [60]. As discussed above, there is a multidimensional effect of social identity on consumers’ value perceptions. Therefore, this study expects to find that consumers’ social identification with green consumer groups positively influences their perceived value of green food. The hypotheses of this study are as follows:

**Hypothesis** **2a (H2a).** *Cognitive identity positively influences the green perceived value.*

**Hypothesis** **2b (H2b).** *Emotional identity positively influences the green perceived value.*

**Hypothesis** **2c (H2c).** *Evaluative identity positively influences the green perceived value.*

#### 2.1.3. Social Identity and Psychological Distance

Psychological distance is a social psychological term that originated from the field of aesthetic philosophy [61]. In the field of social psychology, following the research that was conducted by Liberman et al., psychological distance was developed into a construal-level theory [62]. The theory suggests that as individuals’ psychological distance from perceived people and things decreases, their descriptions of them become more specific and concrete. Conversely, the more psychological distance people believe they have between themselves and other people or events, the more abstract their descriptions are [63]. Psychological distance has since been used across various academic disciplines and has been given varied definitions. Huang stated that psychological distance is a subjective judgment based on the degree of closeness to and acceptance of others [26]. Chen et al., approach psychological distance as a description of the subject’s degree of agreement with the perceived object [64]. In summary, this paper defines psychological distance as the perception of the individual, originating from the self, regarding the degree of separation or proximity to green food. It encompasses the sense of alienation or closeness associated with this perception [25].

Existing research on social identity theory suggests that trust or group identity can lead to reciprocal expectations [65]. Thus, individuals who identify with a group may have a very positive attitude toward the support and programs offered by that group [66]. This suggests that people prefer to trust groups that share their values [67,68]. However, psychological distance is different from social identity. The presence of psychological distance implies a sense of uncertainty in individuals, which leads to distrustful attitudes [69,70]. For example, Safari et al., argue that the increased psychological distance due to cross-border online purchases could be reduced by increasing trust [71], while Cui et al., showed that reducing psychological distance positively affects consumers’ trust in cross-border purchases [72]. To summarize, the trust that arises from social identity has the potential to reduce the psychological distance between consumers and a product. Therefore, this study anticipates that consumers’ social identity with green consumer groups will result in a decreased psychological distance towards green food. The hypotheses of this study are as follows:

**Hypothesis** **3a (H3a).** *Cognitive identity positively influences the psychological distance from green food.*

**Hypothesis** **3b (H3b).** *Emotional identity positively influences the psychological distance from green food.*

**Hypothesis** **3c (H3c).** *Evaluative identity positively influences the psychological distance from green food.*

#### 2.1.4. Green Perceived Value and Purchase Intention

Perceived value refers to consumers’ attitudes toward a product [73]. That is considered to be a summative evaluation of the product [74], and this evaluation guides consumers’ purchase behavior toward the product [75]. Thus, customer perceived value is an important determinant of purchase intention [76,77]. The influence of green perceived value developed from the perceived value on consumer purchase intention has been thoroughly demonstrated by scholars [12,78,79]. Despite the ongoing debate surrounding this influence relationship, this hypothesis has been included in this study to maintain the integrity of the model and ensure continuity in the derivation of hypotheses. The hypothesis of this study is as follows:

**Hypothesis** **4 (H4).** *Green perceived value positively influences green food consumers’ purchase intention.*

#### 2.1.5. Psychological Distance and Purchase Intention

Psychological distance is widely used to explain consumer psychology and is an important antecedent for determining consumer behavior [80]. According to the level of construal theory, psychological distance describes an individual’s interpretation of the degree of psychological proximity to something [81]. Studies have concluded that the farther that distance is, the less accessible and unreliable the information about things will be, and conversely, the information will be more specific and reliable [62,63,82]. Hence, psychological distance plays a crucial role in shaping consumer mood, which in turn influences their purchasing behavior [83,84]. Chung et al., argue that consumers who have a close psychological distance from a brand may have higher purchase intentions for that brand’s products [85]. Zhang et al., and Peter et al., argue that live video adverts and social proximity can increase consumer trust and purchase intentions by bringing them closer to the brand [86,87]. In summary, this study expected that the shorter the psychological distance consumers have from green foods, the clearer their purchase intentions will be. The hypothesis of this study is as follows:

**Hypothesis** **5 (H5).** *Psychological distance of green food positively influences the purchase intention of green food consumers.*

#### 2.1.6. Psychological Distance and Green Perceived Value

Existing research suggests that the shorter the psychological distance of consumers from a brand or product, the better they rate the brand or product [85]. As psychological distance decreases, consumers tend to perceive greater credibility, usability, similarity, and familiarity [67,88]. A review of existing studies found that credibility [89], usability [90], similarity [91,92], and familiarity [93,94] are all important antecedents that influence consumers’ perceived value. Meanwhile, Yang’s research shows that consumers’ perceived value of word of mouth is affected by their psychological distance from the platform that provides word of mouth [27]. Therefore, this study expects that the closer the consumer’s psychological distance is from green food, the higher the green perceived value will be. The hypothesis of this study is as follows:

**Hypothesis** **6 (H6).** *Psychological distance of green food positively influences the green perceived value.*

#### 2.1.7. The Mediation Effect of Psychological Distance and Green Perceived Value

By summarizing the hypotheses presented in the above study, this study concludes that the ways through which consumers identify with green groups based on cognitive, emotional, and evaluative responses can have a positive impact on their psychological distance from green food by increasing trust and minimizing uncertainty (Hypotheses H2a, H2b, and H2c). Moreover, the psychological distance from green food increases consumers’ perception of green value by increasing credibility, usability, similarity, and familiarity (Hypothesis H6), which eventually increases consumers’ purchase intention toward green food. Therefore, this study expects that there has been a serial mediating effect of psychological distance and perceived green value between social identity and the purchase intention of consumers. The hypotheses of this study are as follows:

**Hypothesis** **7a (H7a).** *Green perceived value has a mediating effect between cognitive identity and green food consumers’ purchase intention.*

**Hypothesis** **7b (H7b).** *Green perceived value has a mediating effect between emotional identity and green food consumers’ purchase intention.*

**Hypothesis** **7c (H7c).** *Green perceived value has a mediating effect between evaluative identity and green food consumers’ purchase intention.*

**Hypothesis** **7d (H7d).** *Psychological distance has a mediating effect between cognitive identity and green food consumers’ purchase intention.*

**Hypothesis** **7e (H7e).** *Psychological distance has a mediating effect between emotional identity and green food consumers’ purchase intention.*

**Hypothesis** **7f (H7f).** *Psychological distance has a mediating effect between evaluative identity and green food consumers’ purchase intention.*

**Hypothesis** **7g (H7g).** *Psychological distance and green perceived value has a serial mediating effect between cognitive identity and green food consumers’ purchase intention.*

**Hypothesis** **7h (H7h).** *Psychological distance and green perceived value has a serial mediating effect between emotional identity and green food consumers’ purchase intention.*

**Hypothesis** **7i (H7i).** *Psychological distance and green perceived value has a serial mediating effect between evaluative identity and green food consumers’ purchase intention.*

### 2.2. Theoretical Framework

**Figure 1:**
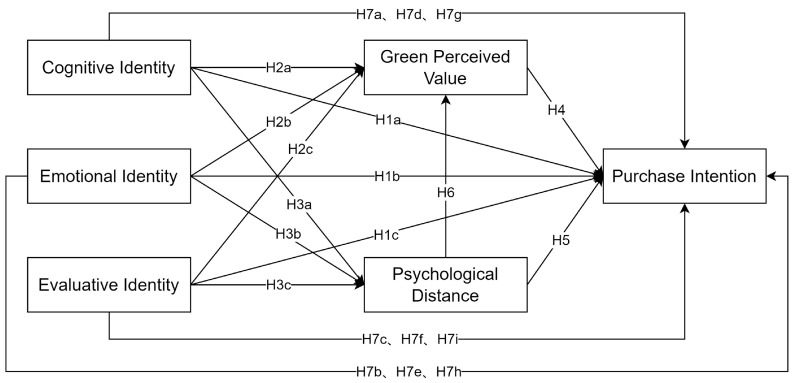
Model of the influence of social identity in green food purchase intention.

This study aims to explore the influence of social identity on the purchase intention of green food. This study combines the analysis of related literature, divides social identity into cognitive, emotional, and evaluative components, and establishes the research model of this paper by combining the hypotheses proposed by the aforementioned studies, as shown in Figure 1.

## 3. Method

### 3.1. Data Acquisition

This study was based on literature analysis, open-ended surveys, multi-person interviews, and communication among experts to develop the final official questionnaire. Before conducting the formal survey, a pilot survey involving 25 consumers was conducted to ensure their comprehension of the variables and questions in the questionnaire. The formal questionnaire was designed using a professional survey website, wjx.cn, and was promoted through social platforms like WeChat and QQ group chats. This approach allowed respondents to conveniently participate in the survey without geographic limitations, using their mobile devices. The questionnaire was distributed in mainland China, with respondents selected based on their green food purchasing experience and age (above 20 years). This age group was chosen because respondents over 20 years old are the primary green consumer demographic in China [95]. The survey was conducted between March and April 2023. Out of the 600 questionnaires distributed, a total of 572 were collected. After excluding 75 questionnaires deemed invalid, the study was left with 497 valid questionnaires. According to Kline’s study [96], a sample size of ten times the number of measurement items is deemed appropriate. In this research a questionnaire with 21 measurement items was utilized, indicating that the 497 collected questionnaires are sufficient and suitable for the next step of data analysis.

### 3.2. Measures

This study conducted a scale selection exercise for the six variables proposed above, as shown in Table 1. The questionnaire design of this paper was adapted from previous studies and combined with the research direction of this paper. All variables were measured using a 7-point Likert scale, divided from 1 (strongly disagree) to 7 (strongly agree).

### 3.3. Data Analysis

SmartPLS4 can offer a wide range of modeling functions and introduces new algorithms. It presents calculation results more clearly in tabular form and provides excellent support for complex models. Therefore, this study used SPSS26 (IBM, Armonk, NY, USA) and SmartPLS4 (SmartPLS GmbH, Oststeinbek, Germany) software to process and analyze the data obtained from the questionnaire. First, the characteristics of the respondents were analyzed using SPSS26. Secondly, SmartPLS4 was used to test the external load coefficient, Cronbach coefficient, CR value, and AVE value. Finally, the research hypothesis was verified using the bootstrapping algorithm, and the model was evaluated using R^2^ and Q^2^ values.

## 4. Results

### 4.1. Respondent Demographic Characteristics

Descriptive statistical analysis was conducted on 497 valid questionnaires. The results are shown in Table 2. According to the analysis results, males accounted for 45.7% of the sample size, and females accounted for 54.3% of the sample size, with the number of male consumers being lower than that of the female consumers, in line with the view that female consumer groups dominate household food consumption [101]. It is also in line with the idea that female consumers constitute the primary group of green consumers [95]. Additionally, the age group of those between 31 and 50 years old accounted for 72% of the sample size, indicating that this group demonstrates a certain level of purchasing power and embraces a relatively novel consumption concept. This also aligns with the predominant age distribution of green consumption in China [95]. What is more, undergraduates accounted for 36% of the sample, and postgraduates accounted for 13.1% of the sample. Most of the participants are social workers, mostly for state-owned companies and private companies, accounting for 33.8% and 44.7%, respectively. Finally, most individuals in this group fall within the range of RMB 3000–9000. The characteristics of the respondents in this study align with the profile of Chinese green consumption consumers, and the sample distribution is sufficiently diverse enabling further research opportunities.

### 4.2. Reliability and Validity Analysis

The results of the reliability and validity tests are shown in Table 3, with Cronbach’s α values ranging from 0.823 to 0.890, both greater than the reference value of 0.7 [102], and CR values ranging from 0.894 to 0.924, both greater than the reference value of 0.6 [103], indicating that the overall internal consistency of the scale is good. The reliability of the scale is guaranteed. PLS-SEM uses external loadings and AVE as metrics to judge convergent validity. The external loadings ranged from 0.821 to 0.916, both of which were greater than the reference value of 0.6 [104], and the AVE values ranged from 0.712 to 0.758, both of which were greater than the reference value of 0.5 [104]. Therefore, it can be suggested that the scales have high validity.

### 4.3. Discriminant Validity Analysis

This paper uses two methods to verify the discriminant validity of the model. The first method is the Fornell–Larcker Criterion, where the criterion is that the correlation coefficient between latent variables should be less than the root value of their own AVE. The second method is HTMT (Heterotrait-monotrait Ratio). It is judged by the criterion that the HTMT value should be lower than 0.85 [105]. The discriminant validity of this paper’s model passes the two aforementioned methodological tests. The outcomes are displayed in Table 4 and Table 5.

### 4.4. Collinearity Analysis

The collinearity diagnosis is the first step in judging the results of the structural model, and the success criterion indicates that when the internal model VIF is less than 5 [106], there is no serious collinearity effect in the data. As shown in Table 6, the VIF values are between 1.258 and 2.187, thus the structural model studied in this investigation passes the collinearity diagnosis.

### 4.5. Path Analysis

**Figure 2:**
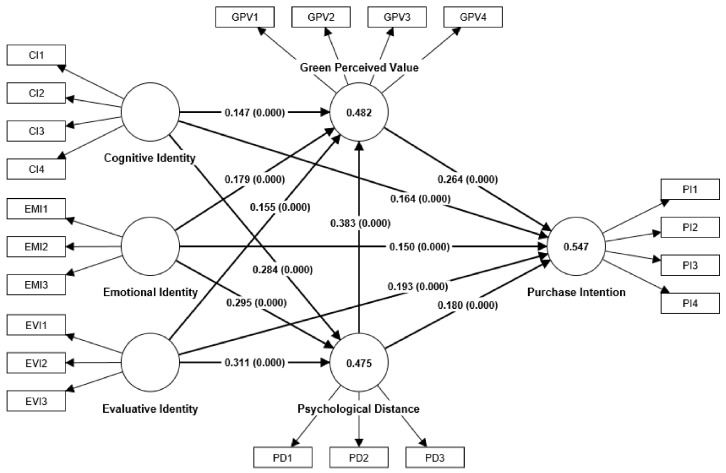
Analytical result of the model.

The structural model path coefficients were evaluated by SmartPLS4, and when the path coefficient *t*-value is greater than 1.96 [104], it means that the path coefficient passed the test at a 5% level of significance, representing a significant path coefficient. The results of the analysis are shown in Table 7 and Figure 2, and according to that, CI (β = 0.164, T = 4.485, *p* = 0.000), EMI (β = 0.150, T = 4.223, *p* = 0.000), and EVI (β = 0.193, T = 4.748, *p* = 0.000) had a significant positive effect on PI, therefore, hypotheses H1a, H1b, and H1c hold. Additionally, CI (β = 0.147, T = 3.805, *p* = 0.000), EMI (β = 0.179, T = 4.412, *p* = 0.000), and EVI (β = 0.155, T = 3.784, *p* = 0.000) had a significant positive effect on GPV, and hypotheses H2a, H2b, and H2c hold. CI (β = 0.284, T = 7.941, *p* = 0.000), EMI (β = 0.295, T = 7.842, *p* = 0.000), and EVI (β = 0.311, T = 8.571, *p* = 0.000) had a significant positive effect on PD, and thus, hypotheses H3a, H3b and H3c hold. GPV (β = 0.264, T = 6.212, *p* = 0.000) had a significant positive effect on PI, hence hypothesis H4 holds. PD (β = 0.180, T = 4.023, *p* = 0.000) had a significant positive effect on PI; therefore, hypothesis H5 holds. PD (β = 0.383, T = 8.831, *p* = 0.000) had a significant positive effect on GPV; consequently, hypothesis H6 holds. What is more, GPV had a mediating effect between CI and PI (β = 0.039, T = 3.211, *p* = 0.001), EMI and PI (β = 0.047, T = 3.518, *p* = 0.000), and EVI and PI (β = 0.041, T = 3.142, *p* = 0.002), and so hypotheses H7a, H7b, and H7c hold. PD had mediating effects between CI and PI (β = 0.051, T = 3.478, *p* = 0.001), EMI and PI (β = 0.053, T = 3.538, *p* = 0.000), and EVI and PI (β = 0.056, T = 3.680, *p* = 0.000), whence hypotheses H7d, H7e, and H7f hold. PD and GPV had serial mediation effects between CI and PI (β = 0.029, T = 4.292, *p* = 0.000), EMI and PI (β = 0.030, T = 4.350, *p* = 0.000), and EVI and PI (β = 0.031, T = 4.365, *p* = 0.000), and thus hypotheses H7g, H7h, and H7i hold.

### 4.6. Model Explanatory and Prediction Ability

R² represents the joint effect of all predictive variables of a latent variable on that latent variable, whereas Q^2^ enables the evaluation of the predictive validity of the model. When the R^2^ value is greater than 0.25 (R^2^ values for GPV, PD, and PI are all greater than 0.25) [107], the model can have strong explanatory power. When the Q^2^ value is greater than 0 (Q² values of GPV, PD, and PI are all greater than 0) [108], the model is eligible for having good predictive ability. All results are shown in Table 8.

## 5. Discussion

Most previous studies on green food and purchase behavior have primarily relied on theoretical models, with limited research utilizing the social identity theory to investigate green food purchase intentions. Therefore, this study explored the effects of the cognitive, emotional, and evaluative components of social identity on green food purchase intentions, as well as the serial mediating effects of green perceived value and psychological distance in the above relationships.

First, cognitive identity, emotional identity, and evaluative identity each positively impact purchase intention. Bartels et al. study on organic food is similar [47], but the difference between this study and the present study is that this study was conducted only from the perspective of the cognitive component of social identity, while the present study was conducted with cognitive, emotional, and evaluative components as entry points. Also, the data show that the evaluative component of social identity has the most significant impact on purchase intentions. This suggests that the evaluative component of consumers’ social identity for green consumer groups is more effective in increasing their purchase intention for green food, compared to the cognitive and emotional components which are brought by self-image, values, lifestyle, praise, attachment, and sense of belonging.

Secondly, according to the results of the study, it seems that the cognitive component, the emotional component, and the evaluative component of social identity can positively impact green perceived values. He et al. found similar results in a study on social identity [57]; yet, their research was primarily focused on examining the impact of social identity on perceived value from the brand identity perspective. In contrast, this study demonstrated the impact of social identity on green perceived value from a multidimensional perspective. This study broadens the research on social identity and perceived value and extends the study of the social identity theory to the field of green food consumption.

Moreover, the results showed that the cognitive, emotional, and evaluative components of identity positively influence psychological distance. Previous studies on social identity and psychological distance have emphasized the influence of social identity on trust and the subsequent impact of trust on psychological distance [66,71]. Additionally, the direct effect of social identity on psychological distance has rarely been verified. This study demonstrates that the greater consumers socially identify with a green consumer group, the greater their affinity for the group’s value will be. Therefore, individuals with a stronger social identity are more likely to exhibit a reduced psychological distance toward green food. According to the data of this study, the evaluative component of social identity is the variable that has the greatest impact on psychological distance. This finding emphasizes that the effect of group self-esteem on psychological distance in social identity exceeds the effect of self-categorization and affective commitment on psychological distance.

In addition, psychological distance and green perceived value positively impact green food consumers’ purchase intention. This can be explained by the consumption value theory, according to which the value provided by goods is the main factor affecting consumers’ choice of a particular product or brand. This result is consistent with many previous studies [12,23,79,109].

What is more, it is observed that psychological distance positively influences the green perceived value. This finding follows the results of a previous study [27], with the difference that it focused on the effect of the perceived value derived from word-of-mouth in response to networks. The data from the study showed that psychological distance was the variable with the highest degree of influence on green perceived value among all variables. This emphasizes the importance of psychological distance in influencing the perceived value of green food. It also indicates the significance of tangible aspects of green food in consumers’ perceptions as the main factor influencing consumers’ purchases, and the influence of tangible aspects on consumers’ perceptions surpasses the impact of social identification with the green consumer group.

Furthermore, there is a mediating effect of psychological distance and green perceived value between social identity and purchase intention, respectively. This extends the application of green perceived value and psychological distance as mediating variables in the model according to previous studies [110,111]. It shows that consumers’ identification with green consumer groups will lead to a reduced psychological distance and higher green perceived value for green food and ultimately enhance their purchase intentions. This validates the importance of psychological distance and green perceived value in green food consumption and enhances theoretical research on green food purchases in the context of social identity.

Finally, we can argue that there is a serial mediating effect between psychological distance and green perceived value between social identity and purchase intention. This finding contributes to the enrichment of the theoretical model focusing on green food and consumer behavior as the primary subjects of study [112]. It shows that the greater the emotional and value significance that green consumer groups assign to individuals about green food is, the more tangible the individuals’ impressions of green food will be, thus the tangible aspects of green food help bridge the psychological distance between consumers and green food, bringing them closer together. Meanwhile, the proximity of psychological distance facilitates an increase in the perceived value of green food, thus influencing individuals’ purchase intention toward green food. This suggests a causal sequence of social identity-psychological distance-green perceived value-purchase intention and emphasizes that the positive emotional and value significance of green consumer groups to individuals is an important premise of this association.

The establishment of this research model holds practical significance for the green food industry and sustainable development. Firstly, in promoting green consumption, the research findings highlight the close relationship between social identity, green perceived value, and psychological distance with the purchase intention of green food. Green food companies can use these factors to effectively promote green food consumption. Secondly, to improve company competitiveness, the research model assists companies in developing more accurate publicity programs and strengthening their connection with consumers, ultimately improving company competitiveness. Thirdly, in alignment with the goal of sustainable development, the study’s results offer guidance for promoting green food consumption, contributing to the advancement of a more sustainable society.

## 6. Conclusions

The research model in this study can predict consumers’ purchase intention of green food. This has several theoretical implications. First, the Social Identity Theory and Psychological Distance Theory were employed to investigate consumers’ intentions to purchase eco-friendly food by examining individual and group relationships. Secondly, the causal sequence relationship of the model explains the interaction between psychological distance and green perceived value in social identity and purchase intention, which deepens the understanding of social identity theory on consumers’ purchase intention in the context of green food consumption. At the same time, this study holds several managerial implications. Firstly, it offers valuable insights into the psychological process of consumers, enabling stakeholders to gain a deeper understanding of consumer needs. This, in turn, facilitates the development of more effective marketing strategies. Secondly, the study provides stakeholders with a fresh perspective from a group-individual standpoint. It emphasizes the significance of reinforcing consumers’ cognitive, emotional, and evaluative identity with the green group, underscoring that this approach is a crucial means of increasing purchase intention among consumers. Combining the above findings, we make the following recommendations for expanding China’s green food consumption market.

First, it is worthwhile to establish communities on popular social media platforms with a specific focus on green consumption as the central theme. By establishing such groups, consumers are drawn to participate actively and form green consumer communities. The process should focus on the quality of design and creativity of content in online communities, as these key factors play a pivotal role in motivating more consumers to contribute and create content. This fosters the development of online communities [113]. Furthermore, official accounts in the community should also focus on the frequency of content posting. Otherwise, frequent posting may have a counterproductive effect [114]. The existence of such communities can make consumers better identify with the green consumer groups and further concretize consumers’ perceptions of green food products, bringing consumers closer to the psychological distance of green food.

Secondly, it is important to enhance the publicity and promotion of green food. The social and private sectors should introduce consumers to the benefits of green food consumption and the differences this has from other types of diet through various advertising methods to strengthen consumers’ perceived value of green food. Additionally, during the process of recruiting an influencer, attention should be paid to their professionalism, similarity to the green food industry, and consistency in promoting green food. These factors significantly impact the effectiveness of the publicity campaign [115].

Thirdly, it is crucial to enhance the purchasing process for consumers when it comes to green food. The social and private sectors can make green food more widely available by establishing online and offline green food advertising, setting green food placement standards, and creating green food logos.

## 7. Limitations and Future Research

This study inevitably has some limitations but also points the way toward future research. Firstly, while purchase intention is crucial for predicting buying behavior, it does not fully capture consumers’ actual purchase behavior. Therefore, future research will incorporate observation or interviews to better understand consumers’ purchasing behavior towards green food. Secondly, this study only conducted online questionnaire surveys in China, and the scope of the questionnaire surveys affects the universality of the results. Therefore, in future research, we aim to conduct online and offline surveys for consumers in more countries to further verify the validity of the model proposed in this study. Thirdly, the concept of green food is relatively broad, and this study did not focus on a specific product category. Thus, it may lead to some influencing factors being overlooked, resulting in biased results. In future research, categorization studies will be conducted for different green food categories.

## Figures and Tables

**Table 1:** Questionnaire items and adoption sources.

Variables	Items	Sources
Cognitive Identity	My personal identity overlaps with that of the green consumer group in terms of perception.	Wang [32] and Okazaki [97]
	My self-image overlaps with the identity of the green consumer group.	
	My values overlap with those of the green consumer group.	
	My lifestyle overlaps with the green consumer group.	
Emotional Identity	Others’ praise of the green consumer group is like a compliment to my ego.	
	I am very attached to the green consumer group.	
	I can integrate into the green consumer group.	
Evaluative Identity	Others respect my connection with the green consumer community.	
	I am integral to the green consumer group.	
	I am valued by the green consumer group.	
Green Perceived Value	Green food has a high level of quality.	Danish et al. [98] and Lin et al. [99]
	Using green food will give a good impression.	
	Using green food is morally right.	
	Green food consumption caused less environmental damage.	
Psychological Distance	I have a better understanding of green food.	Feng et al. [28] and Peter [87]
	I can accept green food.	
	Green food has a very specific place in my mind.	
Purchase Intention	I am willing to learn more about and purchase green food.	Zheng et al. [100] and Liu et al. [80]
	I would like to recommend green food to more people.	
	If I have enough time, energy and money, I am willing to buy green food.	
	I will give priority to green food when I plan to buy food.	

**Table 2:** Respondent demographic characteristics (*n* = 497).

	Items	Frequency	Proportion
Gender	Male	227	45.7%
	Female	270	54.3%
Age (in years)	21–30	64	12.9%
	31–40	187	37.6%
	41–50	171	34.4%
	>51	75	15.1%
Education	High School and below	112	22.5%
	College	141	28.4%
	Undergraduate	179	36%
	Postgraduate	65	13.1%
Job	Civil servants	22	4.4%
	State-owned enterprises	168	33.8%
	Private enterprises	222	44.7%
	Students	64	12.9%
	Other	21	4.2%
Monthly income (RMB/Yuan)	<3000	64	12.9%
	3000–6000	134	27%
	6000–9000	130	26.2%
	9000–12,000	99	19.9%
	>12,000	70	14.1%

**Table 3:** Reliability and validity analysis.

Constructs	Item	Factor Loadings	Cronbach’s Alpha	CR	AVE
Cognitive Identity (CI)	CI1	0.851	0.884	0.920	0.742
	CI2	0.887			
	CI3	0.860			
	CI4	0.848			
Emotional Identity (EMI)	EMI1	0.866	0.833	0.900	0.749
	EMI2	0.865			
	EMI3	0.866			
Evaluative Identity (EVI)	EVI1	0.849	0.840	0.904	0.758
	EVI2	0.905			
	EVI3	0.857			
Green Perceived Value (GPV)	GPV1	0.916	0.890	0.924	0.752
	GPV2	0.836			
	GPV3	0.855			
	GPV4	0.859			
Psychological Distance (PD)	PD1	0.863	0.823	0.894	0.738
	PD2	0.841			
	PD3	0.873			
Purchase Intention (PI)	PI1	0.821	0.865	0.908	0.712
	PI2	0.864			
	PI3	0.853			
	PI4	0.837			

**Table 4:** Discriminant validity (FORNELL).

	CI	EMI	EVI	GPV	PD	PI
Cognitive Identity (CI)	0.861					
Emotional Identity (EMI)	0.340	0.866				
Evaluative Identity (EVI)	0.437	0.418	0.871			
Green Perceived Value (GPV)	0.475	0.494	0.508	0.867		
Psychological Distance (PD)	0.520	0.521	0.558	0.639	0.859	
Purchase Intention (PI)	0.518	0.510	0.562	0.629	0.620	0.844

**Table 5:** Discriminant validity (HTMT).

	CI	EMI	EVI	GPV	PD	PI
Cognitive Identity (CI)						
Emotional Identity (EMI)	0.393					
Evaluative Identity (EVI)	0.507	0.499				
Green Perceived Value (GPV)	0.532	0.573	0.587			
Psychological Distance (PD)	0.608	0.629	0.671	0.747		
Purchase Intention (PI)	0.591	0.601	0.658	0.716	0.734	

**Table 6:** VIF value of the inner model matrix.

	CI	EMI	EVI	GPV	PD	PI
Cognitive Identity (CI)				1.438	1.284	1.480
Emotional Identity (EMI)				1.423	1.258	1.485
Evaluative Identity (EVI)				1.560	1.376	1.606
Green Perceived Value (GPV)						1.929
Psychological Distance (PD)				1.904		2.187
Purchase Intention (PI)						

**Table 7:** Path analysis.

Hypotheses	β	SD	T	*p*	LLCI	ULCI	Decision
H1a: CI→PI	0.164	0.037	4.485	0.000	0.091	0.234	Supported
H1b: EMI→PI	0.150	0.035	4.223	0.000	0.082	0.220	Supported
H1c: EVI→PI	0.193	0.041	4.748	0.000	0.114	0.272	Supported
H2a: CI→GPV	0.147	0.039	3.805	0.000	0.071	0.222	Supported
H2b: EMI→GPV	0.179	0.041	4.412	0.000	0.098	0.261	Supported
H2c: EVI→GPV	0.155	0.041	3.784	0.000	0.074	0.233	Supported
H3a: CI→PD	0.284	0.036	7.941	0.000	0.212	0.354	Supported
H3b: EMI→PD	0.295	0.038	7.842	0.000	0.222	0.369	Supported
H3c: EVI→PD	0.311	0.036	8.571	0.000	0.239	0.381	Supported
H4: GPV→PI	0.264	0.043	6.212	0.000	0.180	0.349	Supported
H5: PD→PI	0.180	0.045	4.023	0.000	0.094	0.268	Supported
H6: PD→GPV	0.383	0.043	8.831	0.000	0.298	0.466	Supported
H7a: CI→GPV→PI	0.039	0.012	3.211	0.001	0.017	0.065	Supported
H7b: EMI→GPV→PI	0.047	0.013	3.518	0.000	0.023	0.076	Supported
H7c: EVI→GPV→PI	0.041	0.013	3.142	0.002	0.018	0.069	Supported
H7d: CI→PD→PI	0.051	0.015	3.478	0.001	0.025	0.082	Supported
H7e: EMI→PD→PI	0.053	0.015	3.538	0.000	0.026	0.084	Supported
H7f: EVI→PD→PI	0.056	0.015	3.680	0.000	0.028	0.087	Supported
H7g: CI→PD→GPV→PI	0.029	0.007	4.292	0.000	0.017	0.043	Supported
H7h: EMI→PD→GPV→PI	0.030	0.007	4.350	0.000	0.018	0.044	Supported
H7i: EVI→PD→GPV→PI	0.031	0.007	4.365	0.000	0.019	0.047	Supported

**Table 8:** R^2^ value and Q^2^ value.

	R^2^	Q^2^ Predict
Green Perceived Value (GPV)	0.482	0.397
Psychological Distance (PD)	0.475	0.468
Purchase Intention (PI)	0.547	0.463

## Data Availability

The data supporting the findings of the current study are available from the corresponding author.
